# Imported Malaria at the Ibn Sina University Hospital in Rabat: A Retrospective Study of 81 Cases

**DOI:** 10.7759/cureus.60253

**Published:** 2024-05-14

**Authors:** Amal Hanafi, Imane Zouaoui, Hasnae Abjabja, Youssef Abercha, Sarra Aoufi

**Affiliations:** 1 Central Laboratory of Parasitology and Mycology, Ibn Sina University Hospital, Faculty of Medicine and Pharmacy, Mohammed V University in Rabat, Rabat, MAR

**Keywords:** morocco, thrombocytopenia, plasmodium falciparum, diagnosis, imported malaria

## Abstract

Background

In parallel with the eradication of indigenous malaria since 2005 and the certification of Morocco as a malaria-free country by the World Health Organization in 2010, imported malaria cases are still being notified in Morocco. This study aims to describe the epidemiological profile and characterize the demographic, clinical, and biological profile of imported malaria cases diagnosed at the Central Laboratory of Parasitology-Mycology of the Ibn Sina University Hospital in Rabat, Morocco.

Methodology

This retrospective study analyzed 81 cases of imported malaria at Ibn Sina University Hospital’s Central Laboratory of Parasitology-Mycology in Rabat, Morocco from January 2015 to December 2023. Patients meeting the inclusion criteria had contracted malaria in endemic regions, confirmed through parasitological evidence on blood smears.

Results

Among the 81 positive cases, 55 (63%) were male, resulting in a male-to-female ratio of approximately 3:1. The imported cases came from 15 countries in sub-Saharan Africa, mainly from Ivory Coast (31 patients, 31%) and Guinea (16 patients, 16%). The main clinical sign was fever (79 patients, 97.53%). The majority of patients (70 patients, 86%) suffered from anemia, while thrombocytopenia was present in 76% of patients (62 patients). *Plasmodium falciparum* was the most common species found in 77 (95%) cases and *Plasmodium ovale* in two (2.5%) cases. However, *Plasmodium vivax* was isolated in only one (1.23%) case. Only one case of co-infection by *P. falciparum* and *Plasmodium malariae* (1.23%) was found. Parasitemia values due to *P. falciparum *were between 0.1% and 30%. On the other hand, those of other species did not exceed 2%.

Conclusions

In summary, among 81 imported malaria cases, 55 (63%) were men, imported mainly from 15 sub-Saharan African countries. *P. falciparum* was the predominant species. Fever was the most common clinical sign, accompanied by high rates of anemia and thrombocytopenia.

## Introduction

Although Morocco was declared a malaria-free country by the World Health Organization (WHO) in 2010, cases of imported malaria, mainly due to *Plasmodium falciparum*, are regularly reported. Imported malaria, occurring in individuals who have stayed in endemic areas, is defined by the presence of malaria signs confirmed by the detection of *Plasmodium* sp. on blood parasitological examinations [1]. Studying these imported cases can provide an overview of global migration patterns and travel-related health risks, particularly as international travel increases.

To describe the epidemiological profile and characterize the demographic, clinical, and biological profile of malaria cases diagnosed at the Ibn Sina University Hospital in Rabat (CHU Rabat), Morocco, we conducted a study at the Central Laboratory of Parasitology-Mycology of CHU Rabat.

## Materials and methods

This is a descriptive retrospective study of 81 cases of imported malaria. This study was conducted at the Central Laboratory of Parasitology-Mycology of the Ibn Sina University Hospital in Rabat, Morocco, from January 1, 2015, to December 31, 2023.

Definitions of imported malaria cases

Imported malaria has been described as an infection acquired in an endemic area by an individual (either a tourist or indigenous native) but diagnosed in a non-endemic country after the development of the clinical disease [2]. The presumptive diagnosis without parasitological proof is mainly based on the notion of a stay in an endemic area with the presence of clinical signs represented by a periodic fever every three days in the event of infection by the species *P. falciparum*, *Plasmodium vivax*, or *Plasmodium ovale *and every four days in case of infection by the species *Plasmodium* *malariae*. Other clinical signs of presumptive diagnosis include headache, vomiting, diarrhea, and splenomegaly. These clinical signs may be associated with hematological abnormalities, including hemolytic anemia and significant thrombocytopenia. The confirmatory parasitological diagnosis includes a blood smear and a thick blood film. The blood smear makes it possible to highlight the different morphological forms (trophozoites, schizont, and gametocytes) which help in the diagnosis of the species. The thick blood film is a reference technique allowing positive diagnosis by concentration. Parasitemia corresponds to the percentage of parasitized red blood cells compared to the total number of red blood cells counted in the 10 fields of the blood smear.

Data collection

A data collection form was used to collect the data from patient records. The data collected included demographic data (e.g., sex, age, country of origin, return from an endemic area, etc.), clinical presentation (e.g., fever, diarrhea, vomiting, headache, etc.), biological results suggesting malaria (platelet number, hemoglobin level), and parasitological examinations (rapid orientation test, blood smear, and thick blood film).

Statistical analysis

Data entry was performed using Microsoft Excel®. The descriptive and statistical analysis was performed using Python® version 3.11. The one-tailed t-test was used to compare the mean platelet levels of our sample against a literature mean of a healthy African population (216 × 10^3^/µL with a 95% reference interval of 128-365) [3]. The statistical significance threshold was defined as p-values <0.05 (our p-value is <0.0001).

## Results

**Figure 1:**
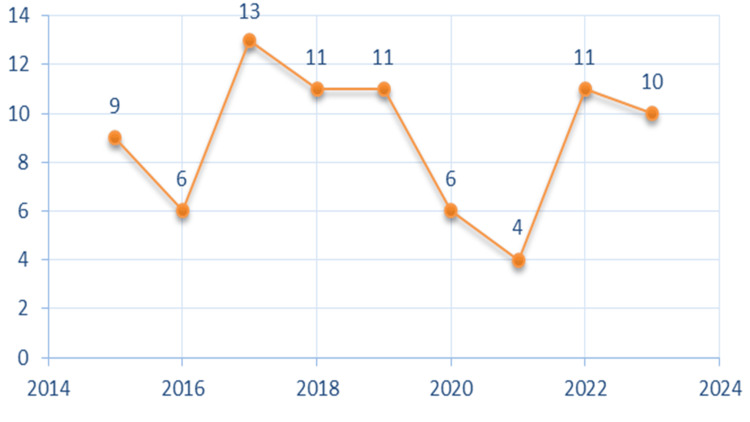
Number of cases of imported malaria declared at the Rabat University Hospital during the period 2015-2023.

A total of 13 (38%) patients diagnosed in 2017 represented the maximum number of annual cases of imported malaria recorded during the nine-year study period. However, the minimum number of annual cases was four cases diagnosed in 2021 (Figure 1), with an average of nine cases per year.

The average age of the patients was 30.2 years, with a minimum age of four years and a maximum age of 53 years. The male sex represented 68% (55 patients) of the cases, with a male/female sex ratio of 3:1.

**Figure 2:**
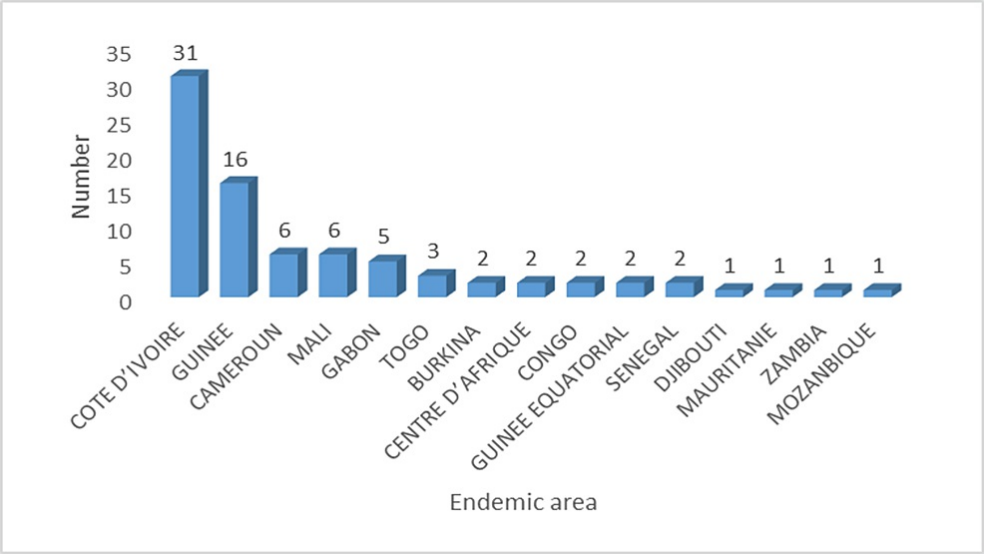
Geographical distribution of imported malaria cases.

The Ivory Coast represented the first endemic malaria transmission country with 38.2% (31 patients) of cases, followed by Guinea with 16% (16 patients) of cases. Other countries, such as Cameroon (six patients, 7.5%), Mali (six patients, 7.5%), Gabon (six patients, 6.1%) and Togo (three patients, 3.5%), were also among these countries (Figure 2). It should be noted that 22 (27%) patients were of Moroccan nationality.

Overall, 54.3% (44 patients) of patients presented to the emergency department, with clinical signs suggestive of malaria 10 days after their return from endemic areas. On the other hand, 38.3% (31 patients) of cases presented between 10 and 30 days of their return, and 6.2% (five patients) of cases within more than 30 days.

**Figure 3:**
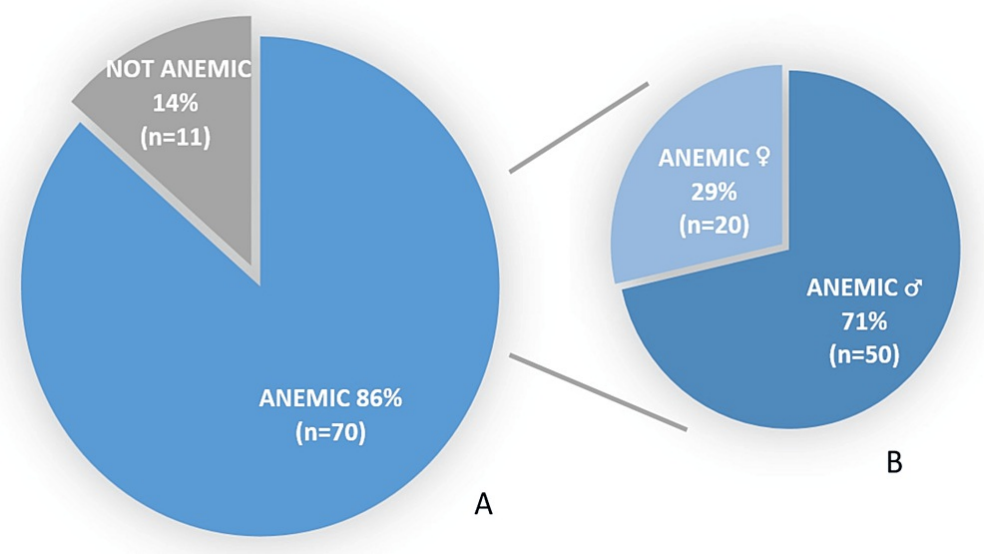
Distribution of cases according to the presence or absence of anemia. A: The percentage of cases. B: The distribution of anemic cases by sex.

Fever (79 patients, 97.53%) was the most common clinical sign. It was associated with headache in 33.33% (27 patients) of patients, vomiting in 28.4% (23 patients) of cases, and diarrhea with abdominal pain in 7.4% (six patients) of cases. Other signs such as pallor, asthenia, jaundice, chills, and splenomegaly were also observed. The number of anemic patients was 70, representing 86% of our series (Figures 3A, 3B). The average hemoglobin level was 11.2 g/dL and the mean corpuscular volume averaged 83 µm^3^ (63.9-103.1 µm^3^), with the majority of patients (83.9%) showing normocytosis. Thrombocytopenia was present in 62 (76%) patients, with a median platelet count of 75.2 × 10^3^/µL.

The confirmatory diagnosis of malaria was based on the analysis of the thick blood film, with an identification of the species performed using a thin blood smear. The orientation test of the direct positive diagnosis by immunochromatographic was negative in four individuals.* P. falciparum* was found in 77 (95%) cases, representing the most predominant species, followed by the *P. ovale* in two (2.5%) cases, and one (1.2%) case by* P. vivax*. One (1.2%) case was identified as a co-infection by *P. falciparum* and *P.* *malariae*.

*P.* *falciparum* parasitemia ranged between 0.1% and 30% in our patients In total, 29 (38.4%) patients had a parasitemia of less than 1%, 21 (27%) patients had a parasitemia between 1% and 4%, and 27 (34.6%) patients had a parasitemia of more than 4% (Table 1). For other *Plasmodium* sp., the *P*. *ovale*, *P. vivax*, and *P. malaria*, parasitemia did not exceed 2%.

**Table 1:** Plasmodium falciparum parasitemia thresholds observed in our study. Parasitemia is the percentage of parasitized red blood cells compared to the total number of red blood cells counted in the 10 fields of the blood smear.

Parasitemia	N (%)
<1%	29 (38.4%)
1–4%	21 (27%)
>4%	27 (34.6%)

## Discussion

According to the WHO, In 2022, the global tally of malaria cases reached 249 million, well above the estimated number of cases before the COVID-19 pandemic and an increase of five million over 2021 [4]. A total of 81 cases of imported malaria were identified, with a peak of 13 (38%) cases in 2017, at the Central Laboratory of Parasitology-Mycology of the Ibn Sina University Hospital in Rabat, over nine years. Although our series is small, this number represents all cases recorded in our hospital between January 1, 2015, and December 31, 2023.

The restrictions due to COVID-19 explain the 60% decrease in cases between 2020 and 2022 with six (7.4%) cases in 2020 and four (4.9%) cases in 2021. A resumption of observed cases was noted in 2022 with 11 (13.5%) cases. The average age of the patients was 30.2 years with a predominance of the male sex. This observation indirectly suggests that the active age group is the most concerned with travel for work reasons or family visits by nationals of endemic countries. This finding has been confirmed by similar studies conducted in other countries [5,6].

Fever was the most frequent clinical sign (79 patients, 97.53%), which is consistent with other studies conducted in Morocco and Italy, which reported the same clinical sign in all patients [7,8]. Signs such as jaundice and splenomegaly associated with more serious complications of malaria and requiring special medical attention have also been reported in the literature in a limited number of patients [9].

Anemia is related to the destruction of erythrocytes during schizogony, increased splenic clearance, and inhibition of erythropoiesis by tumor necrosis factor-alpha and other factors. The exact mechanisms of these processes remain to be elucidated [10,11]. Thrombocytopenia observed in 62 (76%) patients, associated with malaria, usually develops within 24 to 48 hours after infection, progressing in parallel with fever and parasitemia [3,12]. The mechanisms of malaria thrombocytopenia involve the destruction of platelets in the spleen, although these processes remain partially understood [13]. Some studies noted an increase in anti-platelet IgGs, confirming that *Plasmodium*-specific IgGs bind directly to the malaria antigen in platelets, leading to their elimination by phagocytosis in the spleen [14]. Our results are consistent with those reported by other studies which observed a low platelet count in 25 (73.2%) patients and 177 (79%) patients [8,11].

*P. falciparum *was the most isolated species in our study, i.e., 95% (77 patients). According to French and Moroccan studies, percentages of 98% and 66%, respectively, for a population number of 85 and 30 have been reported [6,7]. A high parasitemia rate (>4%) of *P. falciparum* is an indicator of severity, requiring intensive management and follow-up. Our study revealed that 34.6% (27 patients) of cases had a parasitemia greater than 4%. On the other hand, the parasitemia of the other isolated species (*P. ovale*, *P. vivax*, and the co-infection of* P. falciparum* and *P. malaria*) did not exceed 2%, which is in accordance with the reported data [15].

Although the WHO has made antigen tests accessible, the latter remain less sensitive than direct examination methods, which can lead to false negatives even with high parasitemia [9,16]. In our study, the antigen test was negative in four patients.

Studying imported malaria cases is crucial for several reasons. First, it allows us to understand the migratory patterns between countries, particularly as Morocco engages in open relations with other African nations as part of its national policy, fostering reciprocal knowledge exchange and movement in both directions.

Understanding the characteristics of these cases not only contributes to our knowledge of travel medicine but also helps in developing targeted interventions to prevent the spread of malaria across borders. This is especially important given Morocco’s status as a transit hub and its interactions with malaria-endemic regions. Furthermore, comprehending the profile of imported malaria cases is essential for assessing the effectiveness of current preventive measures such as vector control and travel advisories. This understanding aids healthcare professionals in promptly diagnosing and managing cases, thereby reducing malaria-related morbidity and mortality.

## Conclusions

Although there has been an absence of local malaria cases for 17 years, vigilance through enhanced travel surveillance and increased awareness of preventive treatment are essential given the persistence of disease vectors and the possibility of their re-emergence.

The 81 cases of imported malaria, diagnosed at the Central Laboratory of Parasitology-Mycology of the Ibn Sina University Hospital in Rabat from 2015 to 2023, were imported mainly from 15 countries in sub-Saharan Africa. *P. falciparum *was the predominant species. Fever was the most common clinical sign, accompanied by high rates of anemia and thrombocytopenia.
